# Deportalization, Venous Congestion, Venous Deprivation: Serial Measurements of Volumes and Functions on Morphofunctional 99mTc-Mebrofenin SPECT-CT

**DOI:** 10.3390/diagnostics11010012

**Published:** 2020-12-23

**Authors:** Lauranne Piron, Emmanuel Deshayes, Christophe Cassinotto, François Quenet, Fabrizio Panaro, Margaux Hermida, Carole Allimant, Eric Assenat, Georges-Philippe Pageaux, Nicolas Molinari, Boris Guiu

**Affiliations:** 1 Department of Radiology, St. Eloi Hospital, Montpellier University Hospital, 34090 Montpellier, France; c-cassinotto@chu-montpellier.fr (C.C.); m-hermida@chu-montpellier.fr (M.H.); c-allimant@chu-montpellier.fr (C.A.); b-guiu@chu-montpellier.fr (B.G.); 2 Department of Nuclear Medicine, Cancer Institute of Montpellier (ICM), 34090 Montpellier, France; Emmanuel.Deshayes@icm.unicancer.fr; 3 Institute of Research Cancer of Montpellier (IRCM), INSERM U1194, Montpellier University, Cancer Institute of Montpellier (ICM), 34090 Montpellier, France; 4 Department of Surgical Oncology, Cancer Institute of Montpellier (ICM), 34090 Montpellier, France; Francois.Quenet@icm.unicancer.fr; 5 Division of HBP Surgery and Transplantation, Department of Surgery, St. Eloi Hospital, Montpellier University Hospital, 34090 Montpellier, France; f-panaro@chu-montpellier.fr; 6 Department of Oncology, St. Eloi Hospital, Montpellier University Hospital, 34090 Montpellier, France; e-assenat@chu-montpellier.fr; 7 Department of Hepatology and Liver Transplantation, St. Eloi Hospital, Montpellier University Hospital, 34090 Montpellier, France; gp-pageaux@chu-montpellier.fr; 8 IMAG, CNRS, University of Montpellier, Montpellier University Hospital, 34090 Montpellier, France; nicolas.molinari@inserm.fr

**Keywords:** interventional radiology, radionuclide imaging, liver regeneration, hepatectomy, hepatic veins

## Abstract

The objective was to assess the changes in regional volumes and functions under venous-impaired vascular conditions following liver preparation. Twelve patients underwent right portal vein embolization (PVE) (*n* = 5) or extended liver venous deprivation (eLVD, i.e., portal and right and middle hepatic veins embolization) (*n* = 7). Volume and function measurements of deportalized liver, venous-deprived liver and congestive liver were performed before and after PVE/eLVD at days 7, 14 and 21 using 99mTc-mebrofenin hepatobiliary scintigraphy with single-photon emission computed tomography and computed tomography (99mTc-mebrofenin SPECT-CT). Volume and function progressed independently in the deportalized liver (*p* = 0.47) with an early decrease in function (median −18.2% (IQR, −19.4–−14.5) at day 7) followed by a decrease in volume (−19.3% (−22.6–−14.4) at day 21). Volume and function progressed independently in the venous deprived liver (*p* = 0.80) with a marked and early decrease in function (−41.1% (−52.0–−12.9) at day 7) but minimal changes in volume (−4.7% (−10.4–+3.9) at day 21). Volume and function progressed independently in the congestive liver (*p* = 0.21) with a gradual increase in volume (+43.2% (+38.3–+51.2) at day 21) that preceded a late and moderate increase in function at day 21 (+34.8% (−8.3–+46.6)), concomitantly to the disappearance of hypoattenuated congestive areas in segment IV (S4) on CT, initially observed in 6/7 patients after eLVD and represented 35.3% (22.2–46.4) of whole S4 volume. Liver volume and function progress independently whatever the vascular condition. Hepatic congestion from outflow obstruction drives volume increase but results in early impaired function.

## 1. Introduction

Extended hepatic resection with R0–R1 margins is the only way to provide a potential cure to patients with multiple liver tumors. When the future liver remnant (FLR) can be supplied by portal and arterial inflow and drained by at least one hepatic vein (HV), the patient may be eligible for surgery if there is sufficient FLR [1]. Threshold values of acceptable FLR volume to avoid post-hepatectomy liver failure (PHLF) depend on the presumed underlying liver parenchyma, ranging from 20% in healthy livers to 40% in cirrhotic livers [2]. When the FLR is insufficient, preparation of the liver by portal vein embolization (PVE) leads to FLR regeneration [3,4,5] and is the standard of care to obtain an appropriate FLR before surgery [6]. A combination of PVE and embolization of the right and accessory right HVs during the same intervention, called liver venous deprivation (LVD) technique, is used to optimize right PVE results [7]. The same authors also described the extended liver venous deprivation (eLVD) technique including simultaneous embolization of the right portal, right and middle HVs branches, leading to a rapid increase in FLR [8].

Vascular changes occur at a regional level depending on the type of liver preparation used. Indeed, PVE induces right hemiliver (S5-8) deportalization (portal inflow deprivation with preservation of venous outflow) while eLVD induces S5-8 venous deprivation (deprivation of both portal inflow and venous outflow). In addition, eLVD leads to venous congestion of whole or part of the segment IV (S4) due to obstructed venous outflow through the middle HV in a portalized region. Deportalization, venous deprivation and venous congestion after liver preparation represent venous-impaired vascular conditions that have not yet been studied.

Assessment of FLR regeneration is usually based on a volumetric evaluation using computed tomography (CT) at a single point in time 3–4 weeks after liver preparation [4,5]. However, because the FLR volume endpoint depends on the presumed quality of the liver parenchyma, it has recently been suggested that FLR function might be a more valuable tool in predicting PHLF [9]. Dynamic 99mTc-mebrofenin hepatobiliary scintigraphy with single-photon emission computed tomography (SPECT) and CT (99mTc-mebrofenin SPECT-CT) is a quantitative method developed to evaluate total and regional liver function, especially FLR function [10]. Taken up by hepatocytes via organic anion-transporting polypeptides, 99mTc-mebrofenin is directly excreted into the bile canaliculi without undergoing any biotransformation [11]. Although a few studies have evaluated 99mTc-mebrofenin SPECT-CT after PVE [11,12,13,14], to our knowledge, serial measurements of regional liver function have never been reported. Specifically, outflow obstruction of S4 may happen after right hemihepatectomy when the middle HV is harvested for oncological reasons. The evolution of S4 function has never been investigated so far.

The purpose of this study was to assess changes in volume and function in the noncirrhotic liver under venous-impaired vascular conditions induced by liver preparation (deportalization, venous deprivation and venous congestion), using regional serial measurements on morphofunctional 99mTc-mebrofenin SPECT-CT.

## 2. Materials and Methods

### 2.1. Study Design, Patients and Ethics

**Figure 1:**
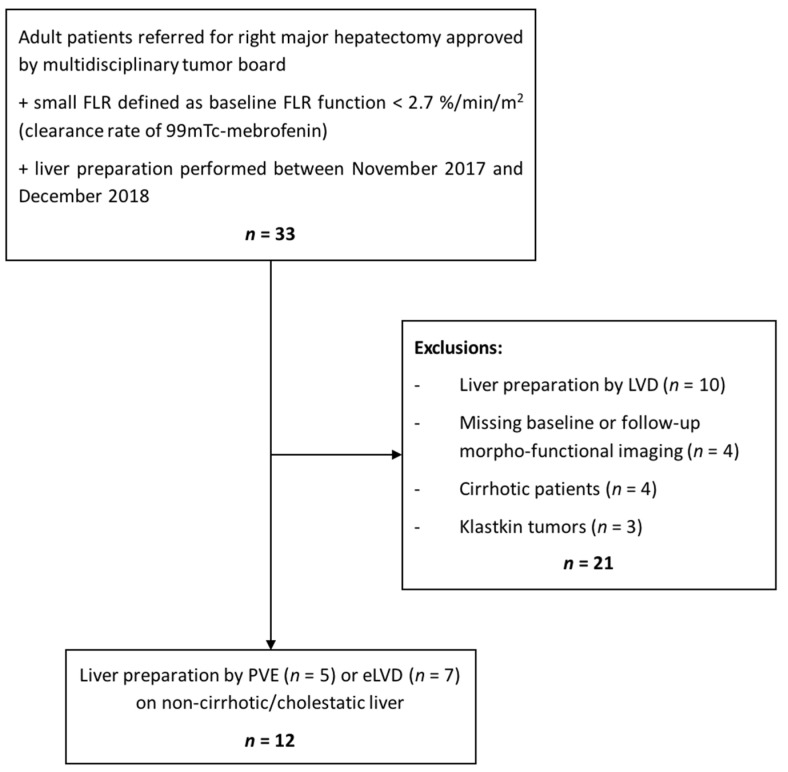
Study flowchart. FLR = future remnant liver; LVD = liver venous deprivation; PVE = portal vein embolization; eLVD = extended liver venous deprivation.

Our study included a retrospective review of a prospective database. The inclusion criteria were as follows: (a) adult patients referred for right major hepatectomy approved by a multidisciplinary tumor board, (b) small FLR defined as baseline FLR function <2.7%/min/m^2^ (clearance rate of 99mTc-mebrofenin) [10], (c) liver preparation performed at our institution between November 2017 and December 2018. The exclusion criteria were (a) liver preparation by LVD, (b) missing baseline or follow-up morphofunctional imaging (i.e., 99mTc-mebrofenin SPECT-CT), (c) patients with cirrhosis and those with biliary obstruction (Klastkin tumors) to avoid bias on impaired liver regeneration. Twelve patients prepared by PVE (*n* = 5) or eLVD (*n* = 7) met the inclusion/exclusion criteria. The flowchart is shown in Figure 1. All patients gave written informed consent. The institutional review board (IRB) approved this retrospective study (project 2019_IRB-MTP_05-15 approved on 2019-06-03 by IRB of Montpellier) (NCT03995459). All data were evaluated anonymously.

### 2.2. Baseline and Follow-Up Morphofunctional Imaging

At baseline (prior to liver preparation) and at days 7, 14 and 21 after PVE/eLVD intervention in all patients, 99mTc-mebrofenin SPECT-CT was performed on a hybrid SPECT-CT scanner (Discovery NM/CT670 pro device, General Electric Healthcare, Milwaukee, USA). After an injection of 150 MBq of 99mTc-mebrofenin (Cholediam, Mediam Pharma, Loos, France), a 6 minute dynamic acquisition was obtained to assess the whole liver clearance rate expressed in %/min/m^2^, normalized for body surface area calculated using the Mosteller formula, as described by Ekman et al. [15]. A fast SPECT acquisition (projection time of 8 s, 60 projections, Matrix 128 × 128) was performed as described by De Graaf et al. [10]. A CT scan acquisition with 2.5 mm image slices was then performed using the same gantry during the portal venous phase, i.e., 70 s after venous administration of 1.5 mL/kg of 400 mgr/mL of iodine (iomeprol, Iomeron 400; Bracco Imaging, Milan, Italy) at 3 mL/s. Volumetrix^®^ Software (GE Healthcare, Milwaukee, MI, USA) was used to reconstruct SPECT data using an iterative algorithm (OSEM) to produce attenuation-corrected images. Coregistration between CT and SPECT images was visually checked and corrected if required since Nivaggioni et al. has shown no impact of manual registration between SPECT and CT on liver functional assessment [16]. Regions of interest (i.e., S5-8 and S4) were created on portal venous phase CT based on anatomical landmarks (falciform ligament as the left border of S4, and the gallbladder and middle HV between S4 and S5-8) with a hand-held cursor by a radiologist (L.P.) with 4 years of experience in liver imaging. The respective volumes were automatically calculated by the workstation (OsiriX MD, Pixmeo, Bernex, Switzerland). Tumor volumes were calculated in a similar manner and regional volumes were defined as (Volume of the region of interest − Tumor volume in the region of interest) and expressed in mL. These volumes of interest (VOI) created on CT were exported to the SPECT attenuation corrected data sets. The VOI were slightly and manually adapted by a nuclear medicine physician (E.D.) with 8 years of experience in liver function evaluations to perfectly fit the SPECT data and VOI contours. Finally, both the radiologist and nuclear medicine physician together agreed on VOI on both modalities. The respective actual amounts of 99mTc-mebrofenin in VOI of regions of interest were calculated and regional functions were defined as ((Total counts in region of interest VOI/Total counts in total liver VOI) × Total liver clearance rate) and expressed in %/min/m^2^.

Variations of both regional volumes and functions at days 7, 14 and 21 were expressed in percentages compared to baseline values.

### 2.3. PVE and eLVD Techniques

All PVE and eLVD interventions were performed under general anesthesia by the same interventional radiologist (B.G.) with 10 years of experience in liver interventional radiology. PVE interventions were performed through a right portal access, using n-butyl-cyanoacrylate (Glubran II, GEM, Italy) and ethiodized oil (lipiodol ultrafluid, Guerbet, Aulnay-sous-bois, France) at a 1:7 ratio. S4 was not embolized. For eLVD interventions, the middle and right HVs (and accessory right HV when present) were embolized percutaneously using oversized Amplatzer Vascular Plugs II (St. Jude Medical, Plymouth, MN), as described by Guiu et al. [8]. *N*-butyl-cyanoacrylate was used for embolization of distal venous HV branches using (Purefill, Peters Surgical, Bobigny, France) and ethiodized oil at a 1:2 ratio.

An abdominal portal venous phase CT (70 seconds after intravenous administration of 1.5 mL/kg of Iomeron 350 at 3 mL/s) was performed at day 1 with a 2.5 mm slice thickness on a 64-detector row Optima 660 multislice CT unit (GE, Milwaukee, WI, USA) to confirm technical success. Hepatic congestion of S4 was evaluated on the same images and defined as portal venous phase hypoattenuation of all or part of S4 [17].

### 2.4. Statistical Analysis

Variations in volume and function from baseline were expressed as medians and interquartile ranges (IQR) at each measurement. A linear mixed model was used to test the independence of variations in volume and function from baseline in the deportalized liver (S5-8 after PVE), the venous-deprived liver (S5-8 after eLVD) and the congestive liver (S4 after eLVD), taking into account repeated measurements over time (at days 7, 14 and 21 after liver preparation). Patients were treated as random factors. The radiologist who performed the first volume measurements repeated those measurements to evaluate intraobserver variability, whereas a second one also performed volume measurements to assess interobserver variability. The nuclear medicine physician repeated function measurements to assess intraobserver variability, whereas a second one also evaluated function measurements to assess interobserver variability. The analyses of each set of data were separated by at least 7 days to prevent any memory bias. Intra- and interobserver correlation and concordance were assessed for both volume and function measurements using Pearson correlation coefficient (r) and Lin concordance coefficient (ρc) [18]. The Lin coefficient combines measures of precision and accuracy to determine whether the observed data deviate significantly from the line of perfect concordance (i.e., the 45° line). All analyses were performed using R software (R Foundation for Statistical Computing, Vienna, Austria). A *p* value < 0.05 was considered to be statistically significant.

## 3. Results

### 3.1. Patient Characteristics at Baseline

Twelve patients (male *n* = 6, female *n* = 6), median age 63 years old (IQR, 56–68), underwent PVE (*n* = 5) or eLVD (*n* = 7) before surgery for colorectal liver metastases (*n* = 11) and hepatocellular carcinoma on histologically-proven healthy liver (*n* = 1). Median BMI was 25.4 kg/m^2^ (IQR, 23.4–29.1). All patients except one (patient 4) underwent a median of 8 cycles of chemotherapy (IQR, 6–12) before preparation of the liver. None of the patients received systemic therapy either the month before or the 3 weeks after liver preparation. There was a technical success in all cases. Patient characteristics at baseline are summarized in Table 1.

### 3.2. Deportalized Liver (S5-8 after PVE)

**Figure 2:**
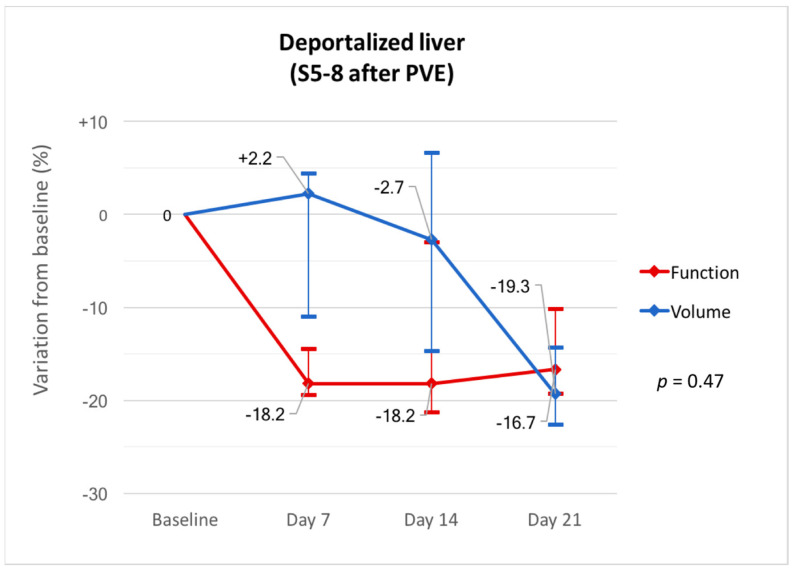
Variations in volume and function from baseline in the deportalized liver (S5-8) at days 7, 14 and 21 after PVE.

Liver volume and function progressed independently in the deportalized liver (*p* = 0.47) with an early decrease in function (median −18.2% (IQR, −19.4–−14.5) at day 7) followed by a decrease in volume (median −19.3% (IQR, −22.6–−14.4) at day 21) (Figure 2). Raw data are summarized in Table 2.

### 3.3. Venous-Deprived Liver (S5-8 after eLVD)

**Figure 3:**
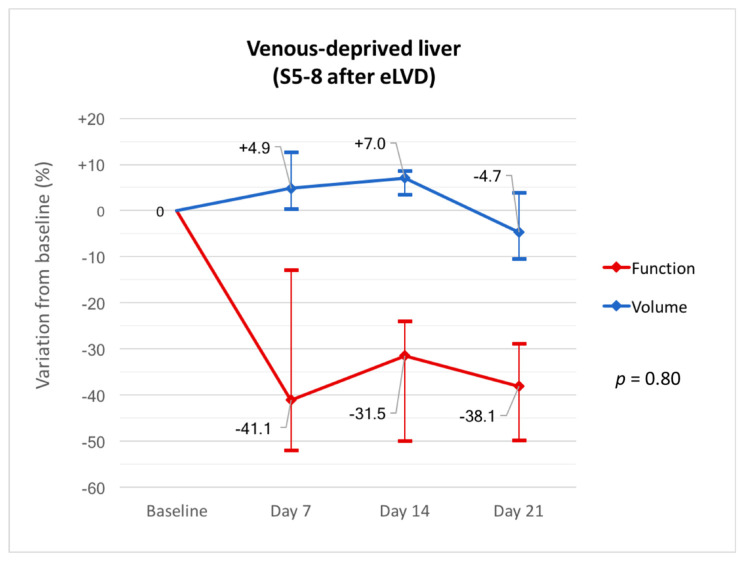
Variations in volume and function from baseline in the venous-deprived liver (S5-8) at days 7, 14 and 21 after eLVD.

Volume and function progressed independently in the venous-deprived liver (*p* = 0.80) with marked differences. There were minimal changes in liver volume in the first 3 weeks (median −4.7% (IQR, −10.4–+3.9) at day 21) while liver function decreased markedly within 7 days after eLVD (median −41.1% (IQR, −52.0–−12.9) at day 7) and remained low thereafter (Figure 3). Raw data are summarized in Table 3.

### 3.4. Congestive Liver (S4 after eLVD)

**Figure 4:**
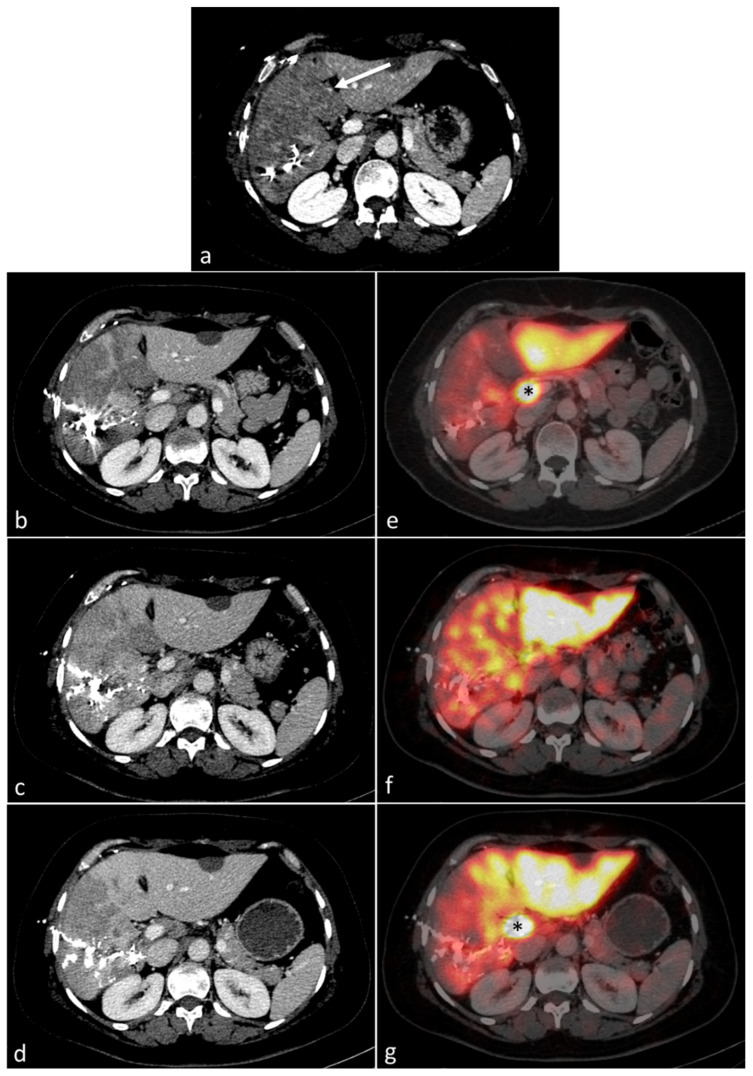
Axial portal venous phase CT at day 1 after eLVD (**a**) showing hypoattenuation in S4 (arrow), which progressively recovers at days 7 (**b**), 14 (**c**) and 21 (**d**). Corresponding 99mTc-mebrofenin SPECT-CT images of the same patient showing progressive increase in S4 function at days 7 (**e**), 14 (**f**) and 21 (**g**). Please note artifactual activity due to the radiotracer accumulation in the main bile duct (black asterisk).

**Figure 5:**
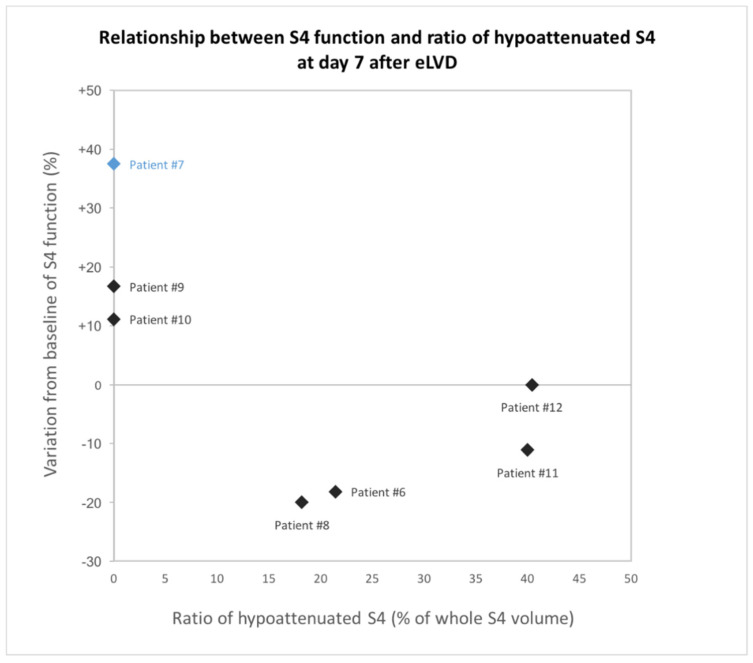
Graph representing the relationship between S4 function and ratio of hypoattenuated S4 on CT in each patient at day 7 after eLVD. The only patient without hypoattenuated area in S4 on CT at day 1 is presented in blue, other patients in black.

**Figure 6:**
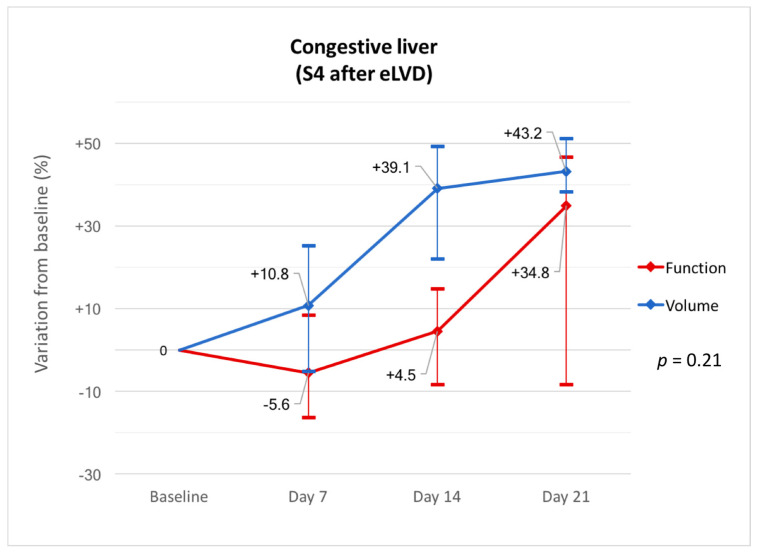
Variations in volume and function from baseline in the congestive liver (S4) at days 7, 14 and 21 after eLVD.

The CT 1 day after eLVD showed hypoattenuated areas representing a median of 35.3% (IQR, 22.2–46.4) of the entire volume of S4 in 6/7 patients. The ratio of congestive S4 gradually decreased thereafter (day 7: median 19.8% (IQR, 4.5–35.3), day 14: median 6.8% (IQR, 0–16.5), day 21: median 0% (IQR, 0–0)) (Figure 4). At day 7, the S4 function in the 4/7 patients with persistent hypoattenuated areas in S4 on portal venous phase CT was decreased or unchanged compared to baseline, while the S4 function in the 3/7 patients without hypoattenuated areas was increased (Figure 5). In particular, the only patient (patient #7) without congestive S4 on CT at day 1 had the greatest increased S4 function at day 7. Further analyses were performed in the six patients with noticeable congestive S4 on CT at day 1. Liver volume and function progressed independently in the congestive liver (*p* = 0.21) and the increase in function was delayed compared to the increase in volume (Figure 6). Indeed, S4 volume increased early and gradually, reaching the maximum increase at day 21 (median +43.2% (IQR, +38.3–+51.2)), while S4 function remained practically unchanged for the first 2 weeks and increased later at day 21 (median +34.8% (IQR, −8.3–+46.6)). Raw data are summarized in Table 4.

### 3.5. Intra- and Interobserver Variabilities

**Figure 7:**
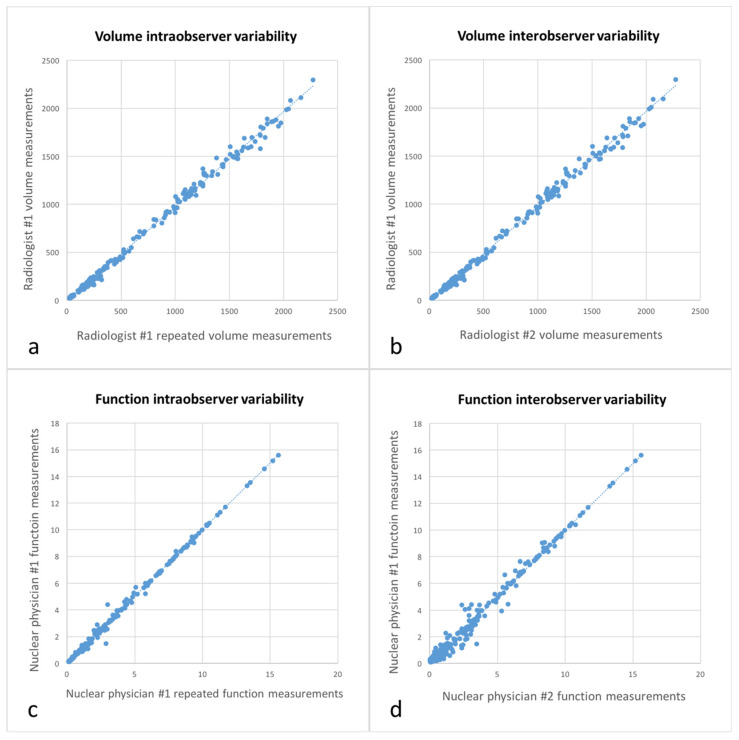
Graphs representing volume intraobserver variability (**a**), volume interobserver variability (**b**), function intraobserver variability (**c**) and function interobserver variability (**d**) with excellent correlation and concordance.

Correlation and concordance were both excellent for volume intraobserver variability (r = 0.998, *p* < 0.001; ρc = 0.998, *p* < 0.001), volume interobserver variability (r = 0.998, *p* < 0.001; ρc = 0.997, *p* < 0.001), function intraobserver variability (r = 0.998, *p* < 0.001; ρc = 0.999, *p* < 0.001) and function interobserver variability (r = 0.993, *p* < 0.001; ρc = 0.993, *p* < 0.001) (Figure 7).

## 4. Discussion

The first important finding of this study is the marked difference in the progression of liver volume and function whatever the vascular condition of the liver. Function decreased markedly in the deportalized liver and even more in the venous-deprived liver as early as day 7 (−41.1% and −18.2% at day 7 after eLVD and PVE, respectively), while volume remained almost unchanged. It has been shown that the embolized liver decreases in volume the first six months after PVE [19]. Although data on LVD/eLVD are limited, atrophy has also been reported in the venous-deprived liver [7,20,21]. Thus, function changes faster than volume. Our results are similar to those in FLR, which show that increase in FLR function is greater than increase in volume at 21–23 days after PVE [12,13,14], suggesting that patients can be safely resected earlier. This marked discrepancy between changes in volume and function has important implications in the evaluation of FLR and suggests that early functional evaluations should be performed using 99mTc-mebrofenin SPECT-CT rather than CT-based volumetry to monitor the increase in FLR and define the appropriate moment for resection. When 99mTc-mebrofenin SPECT-CT is not available for the evaluation of FLR, our results strongly suggest that measurements that do not refer to the embolized volume should be used, such as the standardized FLR volume [22], the FLR volume to body weight ratio [23] or the kinetic growth rate [24].

The second important finding of this study are changes that occur in S4 after outflow obstruction. One out of seven patients did not present with hypoattenuated congestive features on CT and had an early increase in S4 function at day 7 (+37.5%), which was the highest in our series. S4 congestive features were visualized as hypoattenuated areas on CT at day 1 in 6/7 patients as described in previous reports [17,25]. Our consecutive CT scans show gradual disappearance of hypoattenuated areas in S4, which may be due to the development of inter-HV anastomoses leading to outflow redirection towards the left HV, as well as increased lymphatic drainage [26,27]. S4 function only increased at day 7 in patients without hypoattenuation (*n* = 3/7) and decreased or remained stable in the others. Disappearance of S4 hypoattenuation at day 21 was concomitant with the increase in function (+34.8%) in initially congestive S4. Volume and function also progressed independently in the congestive liver. Progressive volume hypertrophy of S4 was observed despite outflow obstruction, leading to a false identification of regeneration of S4. Indeed, S4 function remained nearly unchanged during the first two weeks (−5.6% and +4.5% at days 7 and 14, respectively) with a moderate increase later on (+34.8% at day 21). This suggests that regeneration is impaired in the congestive liver, confirming surgical studies that show reduced S4 regeneration following right hepatectomy with resection of the middle HV [28,29]. This may be due to reversed portal flow in the congestive territories as a result of outflow obstruction [30]. As our study shows, functional recovery following liver congestion takes time (at least 2–3 weeks), which explains the sometimes fatal outcome of PHLF during this period. This is also well known in living donor liver transplantation (LDLT) with the pivotal role of the middle HV. Including middle HV with the right graft can result in impaired remnant liver regeneration with a risk of PHLF in donors [31,32] and remains controversial [33]. Besides LDLT and hemihepatectomy harvesting middle HV, our results are interesting when one HV (usually the middle HV) is ligated or embolized to redirect outflow towards another HV. Nagino et al. first described embolization of the right HV to redistribute outflow towards an inferior-right HV to safely perform left trisegmentectomy with resection of the three major HVs [34]. This concept of outflow modulation may be useful to extend the possibility of resection in complex cases [35,36]. Our results show that although the congestive features (i.e., hypoattenuated areas) resolve within 2–3 weeks, full functional recovery can be longer, thus suggesting that a waiting period of more than 3 weeks is necessary after outflow modulation. This is also true for the optimization of S4 function after eLVD in patients referred for right hemihepatectomy.

Our study has several limitations. First, there were a limited number of patients because we could include only patients prepared using PVE or eLVD, and we had to exclude those having Klatskin tumors or cirrhosis. Patients prepared more recently were enrolled in the HYPERLIV-01 multicenter randomized trial comparing LVD/eLVD to PVE, and thus could not be included in this study. Therefore, our results should be validated in a larger cohort. Moreover, as PHLF generally occurs a few days after liver resection, it would have been interesting to perform 99mTc-mebrofenin SPECT-CT at day 1 to analyze very early function changes. Later functional evaluations (i.e., after 3 weeks) would also have been interesting to further explore changes in function, in particular the recovery of congestive areas. Further prospective studies are needed to explore changes in volume and function over a longer period. Finally, a functional evaluation of hypoattenuated areas in S4 would have been interesting to precisely evaluate the impact of congestion on liver function. However, due to the limited spatial resolution of SPECT, reliable measurements cannot be obtained in these small areas with complex shapes [37].

In conclusion, after preparation of the noncirrhotic liver, liver volume and function progress independently whatever the vascular condition (i.e., deportalization, venous congestion or venous deprivation). Hepatic congestion from outflow obstruction drives volume increase but results in early impaired function that may improve after 2–3 weeks. Morphofunctional 99mTc-mebrofenin SPECT-CT is an attractive imaging modality for liver preparation and surgery, with great potential.

## Figures and Tables

**Table 1:** Patients’ baseline characteristics.

Patient	Intervention	Sex	Age (years)	BMI (kg/m^2^)	Diagnosis	Surgical Resection Planned
1	PVE	F	72	23.4	CRC metastases	RHH + S4
2	PVE	M	59	25.4	CRC metastases	RHH
3	PVE	F	52	20.4	CRC metastases	RHH + S4
4	PVE	F	79	35.5	HCC (healthy liver)	RHH
5	PVE	M	62	29.1	CRC metastases	RHH
6	eLVD	M	65	23.4	CRC metastases	RHH + S4
7	eLVD	F	53	25.4	CRC metastases	RHH + S4
8	eLVD	F	47	20.4	CRC metastases	RHH + S4
9	eLVD	F	66	35.5	CRC metastases	RHH
10	eLVD	M	74	29.1	CRC metastases	RHH
11	eLVD	M	57	23.4	CRC metastases	RHH
12	eLVD	M	65	25.4	CRC metastases	RHH + S1

PVE = portal vein embolization; eLVD = extended liver venous deprivation; F = female; M = male; BMI = body mass index; HCC = hepatocellular carcinoma; CRC = colorectal cancer; RHH = right hemi-hepatectomy; S4 = segment IV according to Couinaud classification; S1 = segment I according to Couinaud classification.

**Table 2:** Volume and function raw data in the deportalized liver (S5-8 after PVE).

Patient	Intervention	Deportalized Liver (S5-8)							
		Volume (mL)				Function (%/min/m^2^)			
		BL	Day 7	Day 14	Day 21	BL	Day 7	Day 14	Day 21
1	PVE	613	535 (−12.7)	523 (−14.7)	525 (−14.4)	9.2	4.9 (−46.8)	5.8 (−37.0)	5.3 (−42.4)
2	PVE	1131	1181 (+4.4)	1100 (−2.7)	875 (−22.6)	8.3	7.1 (−14.5)	8.4 (+1.2)	6.7 (−19.3)
3	PVE	803	821 (+2.2)	899 (+12.0)	648 (−19.3)	10.8	8.7 (−19.4)	8.5 (−21.3)	9.7 (−10.2)
4	PVE	1289	1147 (−11.0)	1090 (−15.4)	908 (−29.6)	3.3	2.7 (−18.2)	3.2 (−3.0)	3.0 (−9.1)
5	PVE	1184	1434 (+21.1)	1262 (+6.6)	1137 (−4.0)	6.6	6.7 (+1.5)	5.4 (−18.2)	5.5 (−16.7)

Data in parentheses are variations from baseline, in %. BL = baseline; PVE = portal vein embolization; S5-8 = right hemiliver (segments V, VI, VII, VIII according to Couinaud classification).

**Table 3:** Volume and function raw data in the venous-deprived liver (S5-8 after eLVD).

Patient	Intervention	Venous-Deprived Liver (S5-8)							
		Volume (mL)				Function (%/min/m^2^)			
		BL	Day 7	Day 14	Day 21	BL	Day 7	Day 14	Day 21
6	eLVD	1003	1021 (+1.2)	1087 (+8.4)	948 (−5.5)	7.9	3.7 (−53.2)	3.7 (−53.2)	3.5 (−55.7)
7	eLVD	705	1017 (+44.3)	801 (+13.6)	672 (−4.7)	6.4	5.4 (−15.6)	4.8 (−25.0)	4.9 (−23.4)
8	eLVD	993	1080 (+8.8)	1080 (+8.8)	1090 (+9.8)	5.7	2.8 (−50.9)	2.9 (−49.1)	3.1 (−45.6)
9	eLVD	1126	1024 (−9.1)	915 (−18.7)	924 (−17.9)	3.9	3.5 (−10.3)	3.0 (−23.1)	3.1 (−20.5)
10	eLVD	1175	1232 (+4.9	1254 (+6.7)	1381 (+17.5)	6.3	2.6 (−58.7)	3.1 (−50.8)	2.9 (−54.0)
11	eLVD	1152	1343 (+16.6)	1233 (+7.0)	1129 (−2.0)	7.3	4.3 (−41.1)	5.0 (−31.5)	4.8 (−34.2)
12	eLVD	1267	1254 (−1.0)	1269 (+0.2)	1072 (−15.4)	4.2	4.1 (−2.4)	3.3 (−21.4)	2.6 (−38.1)

Data in parentheses are variations from baseline, in %. BL = baseline; eLVD = extended liver venous deprivation; S5-8 = right hemiliver (segments V, VI, VII, VIII according to Couinaud classification).

**Table 4:** Volume and function raw data in the congestive liver (S4 after eLVD).

Patient	Intervention	Congestive Liver (S4)							
		Volume (mL)				Function (%/min/m^2^)			
		BL	Day 7	Day 14	Day 21	BL	Day 7	Day 14	Day 21
6	eLVD	209	187 (−10.5)	190 (−9.1)	287 (+37.3)	1.1	0.9 (−18.2)	1.2 (+9.1)	1.5 (+36.4)
7	eLVD	155	237 (+52.9)	244 (+57.4)	238 (+53.5)	0.8	1.1 (+37.5)	1.4 (+75.0)	1.2 (+50.0)
8	eLVD	214	319 (+49.1)	321 (+50.0)	311 (+45.3)	1.0	0.8 (−20.0)	1.0 (0)	0.4 (−60.0)
9	eLVD	168	198 (+17.9)	249 (+48.2)	237 (+41.1)	0.6	0.7 (+16.7)	0.7 (+16.7)	0.8 (+33.3)
10	eLVD	110	101 (−8.2)	143 (+30.0)	175 (+59.1)	0.9	1.0 (+11.1)	1.5 (+66.7)	1.7 (+88.9)
11	eLVD	145	185 (+27.6)	217 (+49.7)	222 (+53.1)	0.9	0.8 (−11.1)	0.8 (−11.1)	0.7 (−22.2)
12	eLVD	191	198 (+3.7)	228 (+19.4)	214 (+12.0)	0.4	0.4 (0)	0.2 (−50.0)	0.6 (+50.0)

Data in parentheses are variations from baseline, in %. BL = baseline; eLVD = extended liver venous deprivation; S4 = segment IV according to Couinaud classification.
